# ER stress links aging to sporadic ALS

**DOI:** 10.18632/aging.101705

**Published:** 2019-01-05

**Authors:** Danilo B. Medinas, Felipe Cabral-Miranda, Claudio Hetz

**Affiliations:** 1 Biomedical Neuroscience Institute, Faculty of Medicine, University of Chile, Santiago, Chile; 2 Center for Geroscience, Brain Health and Metabolism, Santiago, Chile; 3 Program of Cellular and Molecular Biology, Institute of Biomedical Sciences, University of Chile, Santiago, Chile; 4 Buck Institute for Research on Aging, Novato, CA 94945, USA; 5 Department of Immunology and Infectious Diseases, Harvard School of Public Health, Boston, MA 02115, USA; 6 Instituto de Ciências Biomédicas, Universidade Federal do Rio de Janeiro, Rio de Janeiro, Brazil; * Equal contribution

**Keywords:** amyotrophic lateral sclerosis, wild-type SOD-1, aging, ER stress, protein aggregation

Most cases of neurodegenerative diseases such as Alzheimer’s, Parkinson’s, and amyotrophic lateral sclerosis (ALS) have no familial history, being considered sporadic [1]. Aging is the main risk factor to the occurrence of such conditions, which are collectively termed protein misfolding disorders (PMDs) due to the accumulation of aggregated proteins in the brain [2]. Proteostasis impairment is one of the hallmarks of aging, which may contribute to the accumulation of misfolded proteins in disease states. Thus, it is fundamental to understand how age-related alterations to the proteostasis network drive formation of toxic protein species and downstream pathogenic cascades. We reason that profiling the protein misfolding and aggregation process during aging at the biochemical level may uncover molecular pathways underlying disease etiology.

ALS is marked by loss of motoneurons, leading to muscle paralysis and premature death [3]. Mutations in superoxide dismutase 1 (SOD1) cause familial ALS [3], whereas a feverous debate exists over the involvement of wild-type SOD1 (SOD1^WT^) in most common sporadic ALS (sALS) pathology [4]. In this context, we have undertaken a systematic analysis to determine the effects of aging on SOD1^WT^ misfolding and aggregation in mice [5]. The biochemical fingerprinting of young, middle-aged and old animals determined that disulfide-crosslinked SOD1^WT^ aggregates of high molecular weight build-up earlier during aging when compared to other abnormal species (identified by distinct biochemical properties), which may underlie motoneuron vulnerability. Favoring this hypothesis, such aggregates were augmented in post-mortem tissue of sALS patients, supporting their involvement in the pathology.

We then investigated subcellular distribution of SOD1^WT^ aggregates and found that localization to the endoplasmic reticulum (ER) greatly favors protein aggregation through disulfide bonds, which could be prevented by overexpression of chaperones of the protein disulfide isomerase (PDI) family. The accumulation of misfolded and aggregated proteins in the ER lumen generates a stress state, that if chronically sustained, may result in cell demise [6]. ER stress activates the unfolded protein response (UPR), a signaling transduction pathway aimed at restoring ER proteostasis or eliminating irreversibly damaged cells [6]. Signs of ER stress are reported in animal models and patient-derived tissue of several neurodegenerative diseases [7]. In our analysis of patient samples, we corroborated that dysregulation of PDI family members constitutes an important marker of ER proteostasis disturbance in ALS [5,7].

Importantly, ER stress was detected in aged animals, which was even more prominent in SOD1^WT^ transgenic mice. This correlation led us to investigate whether ER stress could trigger the generation of abnormal SOD1^WT^ species. To this aim, we established a drug-induced paradigm of chronic ER stress recapitulating pathological conditions in mice. Remarkably, ER stress specifically induced disulfide-crosslinked SOD1^WT^ aggregates mirroring the effects of aging. At the molecular level, we found that oxidation of a single tryptophan residue (W32) predisposes human SOD1^WT^ aggregation in the ER. Relevantly, W32 had been previously implicated in prion-like propagation of misfolded forms of SOD1 [4]. It remains to be determined whether such disulfide-crosslinked SOD1^WT^ aggregates can be transmitted from cell-to-cell. Furthermore, whether SOD1^WT^ aggregation occurs in motoneurons or glia cells warrants clarification. Noteworthy, the accumulation of SOD1^WT^ aggregates was accompanied by marked astrogliosis, a cell non-autonomous mechanism contributing to motoneuron demise [3].

**Figure 1:**
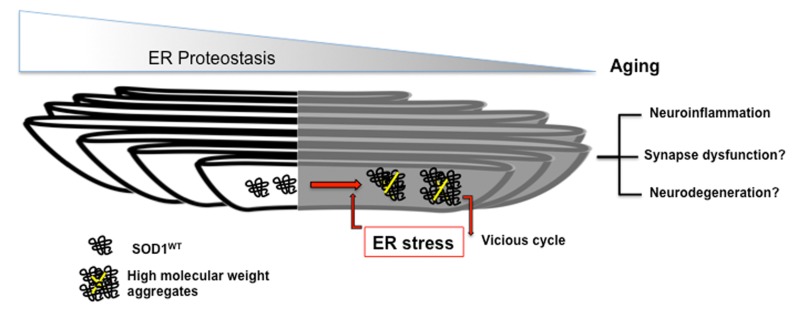
Schematic representation of feed-forward cycle of SOD1^WT^ aggregation and ER stress occurring during aging. ER stress triggers accumulation of disulfide-crosslinked SOD1^WT^ aggregates in the ER of high molecular weight, which further induce ER stress contributing to sALS pathology.

Overall, our study established that ER stress causes SOD1^WT^ aggregation, possibly contributing to sALS etiology through a feed-forward cycle that is exacerbated during aging (Figure 1). Despite the strong evidence linking ER stress to neurodegenerative diseases and aging itself, several fundamental questions await to be answered. To what extent ER proteostasis impairment impact organism life and health span? Why does the ER machinery fail with aging? Does UPR signaling determine neuronal vulnerability in age-related diseases? Finally, do strategies that restore ER proteostasis improve healthspan? The answer to these questions should shed some light on development of therapeutic interventions to neurodegenerative diseases.
